# Comparison of two swept-source optical coherence tomography devices, a Scheimpflug camera system and a ray-tracing aberrometer in the measurement of corneal power in patients with cataract

**DOI:** 10.1007/s00417-023-06348-y

**Published:** 2023-12-27

**Authors:** Shan Ma, Rongyu Gao, Jing Sun, Jun Yang, Kai Wen, Xiteng Chen, Fangyu Zhao, Xinyan Xu, Fang Tian

**Affiliations:** 1 Tianjin Key Laboratory of Retinal Functions and Diseases, Tianjin Branch of National Clinical Research Center for Ocular Disease, Eye Institute and School of Optometry, Tianjin Medical University Eye Hospital, Tianjin, 300380 China; 2 Weifang Eye Hospital, National Key Clinical Specialty, Zhengda Guangming Eye Group, Weifang, 261000 China; 3 Weifang Eye Institute, Weifang, 261000 China

**Keywords:** Corneal curvature, IOLMaster 700, CASIA2, Pentacam, iTrace, Astigmatic vector analysis

## Abstract

**Purpose:**

To assess the differences and similarities in the corneal curvature obtained by two swept-source optical coherence tomography (SS-OCT) devices, Scheimpflug imaging system and one ray tracing aberrometer in patients with cataracts. Moreover, this study aimed to compare the differences in posterior corneal (PK), total corneal (TK) and true net power (TNP) measurements among the IOLMaster 700, CASIA2, and Pentacam.

**Methods:**

A total of 200 eyes of 200 patients (116 female, 58%) were enrolled in this study, with a mean age of 65.9 ± 9.5 years. The flattest (Kf), steepest (Ks), and mean cornal powers (Km), J_0_, and J_45_ were obtained using two SS-OCT-based biometric devices, one rotating camera system and one ray-tracing aberrometer. The PK, TK and TNP values were also measured using these devices. To evaluate the differences and similarities between the devicves, the Friedman test, Pearson correlation coefficient (r), intraclass coefficient correlation (ICC) and Bland‒Altman plots with 95% limits of agreement (LoA) were used, and boxplots and stacked histograms were generated to describe the distributions of the data.

**Results:**

There were no significant differences between the IOLMaster 700 and Pentacam for any of the keratometry values. Additionally, there were no significant differences between the IOLMaster 700 and iTrace in evaluating J_0_ and J_45_. Bland‒Altman plots revealed relatively wide LoA widths, almost larger than 1 diopter for the keratometry values and almost larger than 0.5 diopter for J_0_ and J_45_ values among the four devices. In terms of PK and TK values, significant differences and low ICCs were found among the three devices.

**Conclusions:**

Although strong correlations and good agreement were found among the IOLMaster700, CASIA2, Pentacam and iTrace for Kf, Ks, Km and J_0_, J_45_, it seems that the measurements should not be used interchangeably because of the wide LoA widths and the presence of significant differences among the devices. Similarly, due to significant differences and low ICCs, the PK, TK and TNP values obtained by IOLMaster 700, CASIA2, and Pentacam should not be used interchangeably.

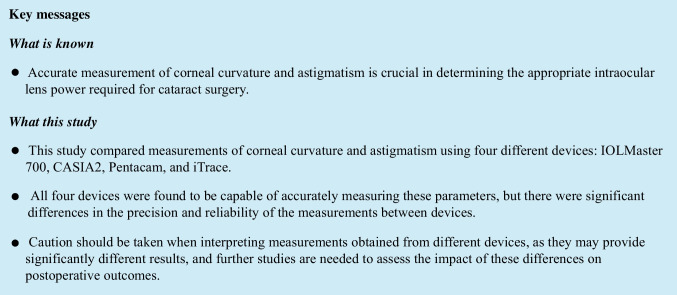

## Introduction

The ever-increasing precision of modern optical biometry, the newer generation intraocular lens (IOL) formula and the ongoing incremental improvements in related technologies and surgical techniques have paved the way for ideal postoperative refractive results and highly anticipated patient expectations undergoing cataract surgery [1]. Hence, the measurement of ocular biometric parameters has become one of the most important practices to precisely assess the eye both for clinical diagnosis and eye surgery [2], especially for cataract operations. This includes not only the anterior corneal curvatures, but also the posterior corneal (PK), total corneal (TK) and true net power (TNP) measurements, which are also considered crucial in achieving accurate refractive outcomes.

In recent years, an increasing number of optical ocular biometers based on different optical technologies and principles for improving the accuracy of ocular measurements have become commercially available. The more recent optical techniques include partial coherence interferometry (PCI), optical low coherence reflectometry (OLCR), optical low coherence interferometry (OLCI), and most recently, swept source-optical coherence tomography (SS-OCT) [3]. The increased life expectancy of the worldwide population has been accompanied by an increase in the pursuit of perfect postoperative refractive visual quality expectations [4], especially for cataract patients, who have a higher desire for glasses independence after surgery. In other words, in addition to routine measurement, the evaluation of corneal parameters with wavefront analysis, including PK, TK and TNP, plays an important role in the preoperative assessment in cataract surgery, especially in eyes that have had prior refractive corneal surgery and in patients who desire a premium IOL [5]. Many systems with different principles, such as Hartmann–Shack, Tscherning, ray tracing (e.g., iTrace) and automated retinoscopy [6], have been widely used in clinical settings in recent years.

Keratometry (K), one of the most indispensable parameters in the calculation of IOL power, can be collected with all the techniques mentioned above, as well as various topographic and tomographic methods, including slit-scanning topography, Placido disc-based keratoscopy, Scheimpflug camera system and optical coherence tomography [7]. As different devices measure keratometry and the PK, TK and TNP values differently, to our knowledge, there is no standard technique for measuring keratometry. Since corneal curvature influences the degree of refraction and high corneal astigmatism may cause a range of visual problems, evaluations of both corneal curvature and corneal astigmatism are essential for cataract surgery [8]. In clinical practice, surgeons must understand whether these devices can be used interchangeably when measuring corneal parameters. There is a paucity of studies exploring anterior keratometry and astigmatism vectors, and PK, TK and TNP values obtained using these devices. Therefore, the purpose of our study was to evaluate the differences and agreements among the SS-OCT-based IOLMater700 and CASIA2, the Pentacam Scheimpflug imaging system and the iTrace ray-tracing aberrometer in the measurement of keratometry and astigmatism vectors, and PK, TK and TNP values.

## Methods

### Study design

This prospective and observational study adhered to the tenets outlined in the Declaration of Helsinki and received approval from the hospital's ethics committee. Informed consent was obtained from all patients, and they were provided with an explanation of the nature of the study before assessments were conducted.

### Patients

Between April and May 2023, two hundred patients (200 eyes; mean age 65.9 ± 9.5 years) who were scheduled for cataract surgery were enrolled in the study. Patients who could not cooperate or were unable to fixate on the internal signal as well as those with a history of eye trauma or previous surgery were excluded from participation. If both eyes of a patient were eligible, one eye was randomly selected. Before surgery, all eyes underwent a complete ophthalmological examination followed by ocular biometric measurements using four devices: an IOLMaster 700 (Carl Zeiss Meditec AG, Jena, Germany), a CASIA2 (Tomey Corporation, Nagoya, Japan), a Pentacam (Oculus, Wetzlar, Germany), and an iTrace (Tracey Technologies Corp., Houston, TX, USA).

### Instruments and measurement

Routine preoperative measurements were taken using the SS-OCT-based IOLMaster 700, CASIA2, Scheimflug imaging system, and aberrometer. Keratometric values, including corneal keratometry (K) measurements at the flat (Kf) and steep (Ks) axes, as well as the mean value (Km), were obtained. Additionally, the posterior corneal (PK), total corneal (TK) and true net power (TNP) values were measured. Corneal astigmatism was transformed into power vectors J_0_ and J_45_, which are better suited to mathematical and statistical analysis as independent, orthogonal components [9]. Two experienced operators measured the parameters using the four devices in random order under the same conditions. Before each measurement, the subject was instructed to place their chin on the chinrest and forehead against the strap, blink completely as needed, and then fixate on the signal target. Only measurements with results indicating "OK" for quality were included in the analysis.

The IOLMaster 700, with a wavelength of 1055 nm and an axial resolution of 22 µm, is an SS-OCT–based device that provides full-eye length tomography that depicts the anatomical details on a longitudinal section through the entire eye. This device measures the anterior surface using eighteen points of telecentric keratometry at 1.5 mm, 2.5 mm, and 3.5 mm; the data from the diameter at 2.5 mm are used for IOL power calculation [1].

The CASIA2 is an anterior segment SS-OCT system that utilizes a 1310 nm wavelength and provides a new method for evaluating corneal morphology. With an axial resolution of < 10 µm, it calculates keratometry values at a diameter of 3.2 mm. Furthermore, it offers measurements for posterior keratometry (PK), and real keratometry, which are employed as total keratometry (TK) in this study. For the CASIA2, TNP was calculated by formula as follows: TNP = K_anterior_ + K_posterior_-[d/(1.376 × 10^6^)] × K_anterior_ × K_posterior_, K_anterior_ and K_posterior_ indicates the refractive power of the anterior and posterior cornea, “d” indicates the cornea thickness (µm).

The Pentacam topography device, consisting of a slit illumination system and a rotating camera with an axial resolution of 50 µm, is currently one of the most used clinical methods for measuring a variety of corneal parameters. In our study, we selected the simulated keratometry (SimK, *n* = 1.3375, 15°), total corneal refractive power (TCRP) values for Kf, Ks, Km, and true net power (TNP) (zone, apex, 3mm) for analysis. Notably, the TCRP values were employed as the total keratometry (TK) in our investigation.

The iTrace system, which uses a combination of Placido corneal topography and a ray‑tracing aberrometer, delivers information about refractive, wavefront and corneal topographic data of the human optical system. It uses 256 parallel thin beams with a wavelength of 785 nm in separate and concentric arrays projected sequentially onto the eye through the pupil [10]. In this study, the keratometry in the central 3 mm zone was studied.

For the astigmatism assessment, vector analysis was performed according to Thibos et al. [11] Double angle plots were created [12]. The power vectors (J_0_ and J_45_) were used to analyze astigmatism, calculated as follows for comparison in a Cartesian coordinate system: J_0_ =  − (C/2) × cos (2 × axis) and J_45_ =  − (C/2) × sin (2 × axis), where C is the negative cylinder (flattest meridian – steepest meridian) and axis is the axis along the flattest meridian. J_0_ represents the vertical (90°)/horizontal (180°) astigmatic component, while J_45_ represents the oblique astigmatic component (45° and 135°).

### Statistical analysis

Statistical analysis was performed using SPSS software (version 25.0; IBM, Armonk, NY) and MedCalc software (version 15.2.2, MedCalc, Ostend, Belgium). All continuous variables were assessed for normality of distribution using the Shapiro–Wilk test and normality assessment plots. Normally distributed data are presented as the mean ± standard deviation (SD), whereas nonnormally distributed data are displayed as the median with interquartile range.

The Friedman test was used to evaluate the differences among the four devices, and Bonferroni adjustment was used for post hoc pairwise comparisons between pairs of devices. Pearson correlation coefficients (r), the intraclass correlation coefficient (ICC), and 95% confidence intervals (95% CIs) were calculated based on a mean-rating, absolute-agreement, two-way mixed-effects model to reflect the correlations and agreements between the measurements. The between-instrument agreement in estimating Kf, Ks, Km, J_0_ and J_45_ was analyzed by the Bland‒Altman method. The 95% limits of agreement (LoA) for each comparison (mean ± 1.96 SD) were used, and the difference between the measurements obtained with the two devices was plotted against their mean. In addition, the width of the LoA was also incorporated in the analysis. A *P* value < 0.05 was considered statistically significant.

## Results

In this study, a total of 200 eyes (118 right eyes) from 200 patients were included in the analysis. The mean age of the participants was 65.9 ± 9.5 years (range: 33–87 years), and 116 (58%) of them were women. The mean AL measured by IOLMaster 700 was 23.79 ± 1.39 mm (range, 21.71–31.00 mm) (Table 1).

**Table 1:** Demographic and biometric data

Demographics	Variables	Range
Patients (eyes), n	200 (200)	−
Age (years) (Mean ± SD)	65.9 ± 9.5	33–87
Sex, female, n (%)	116 (58)	−
Right eye, n (%)	118 (59)	−
AL (mm) (Mean ± SD)	23.79 ± 1.39	21.71–31.00

*SD* standard deviation, *AL* axial length

**Fig. 1:**
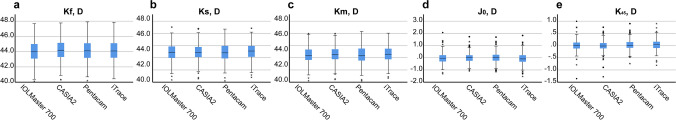
Box plots showing corneal and astigmatic power distributions for IOLMaster 700, CASIA2, Pentacam and iTrace. a: Kf, b: Ks, c: Km, d: J_0_, e: J_45_. The horizontal line within the box indicates the median, and the boundaries of the box indicate the 25th and 75th percentiles. Moderate outliers are marked with asterisks (_*_), and extreme outliers are marked with solid circles (•)

**Fig. 2:**
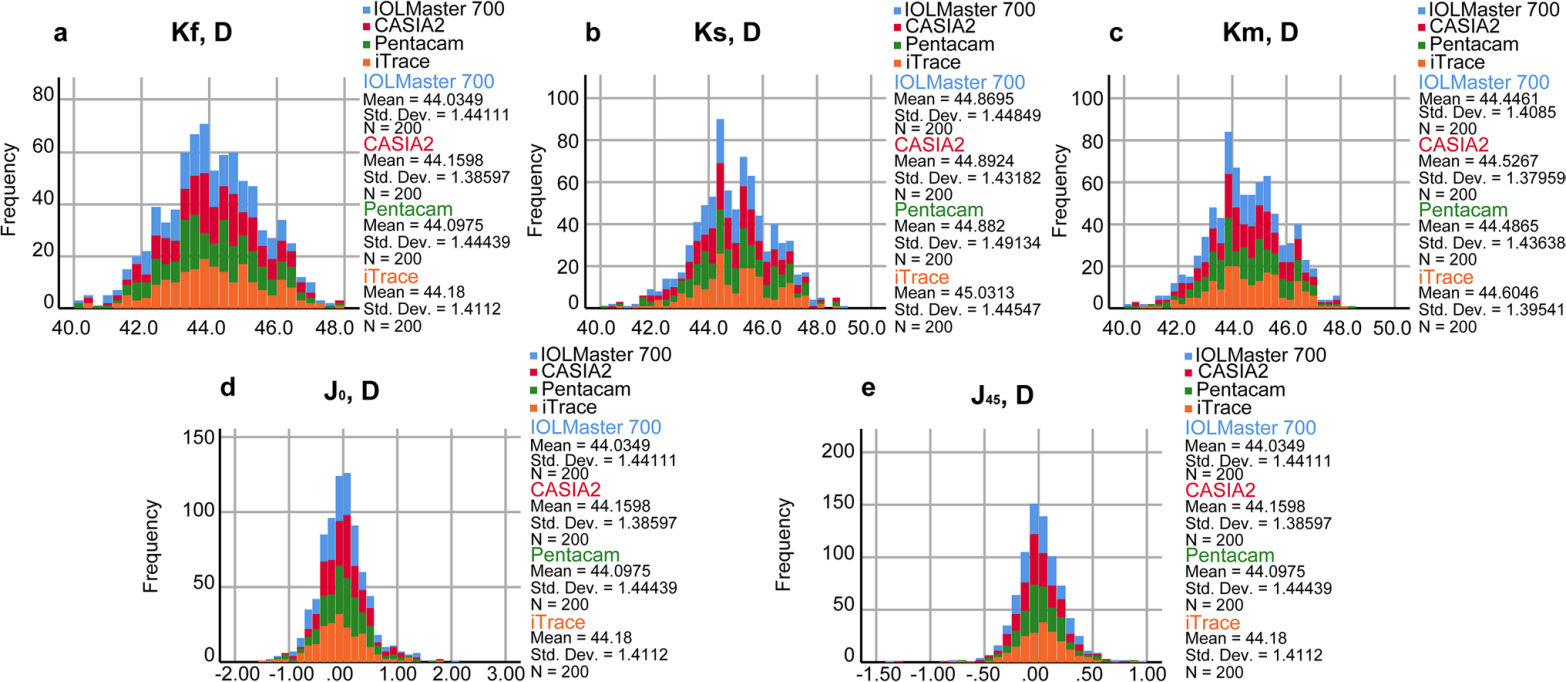
Stacked histogram showing the distribution characteristics of different corneal curvatures for different devices. a: Kf, b: Ks, c: Km, d: J_0_, e: J_45_

**Fig. 3:**
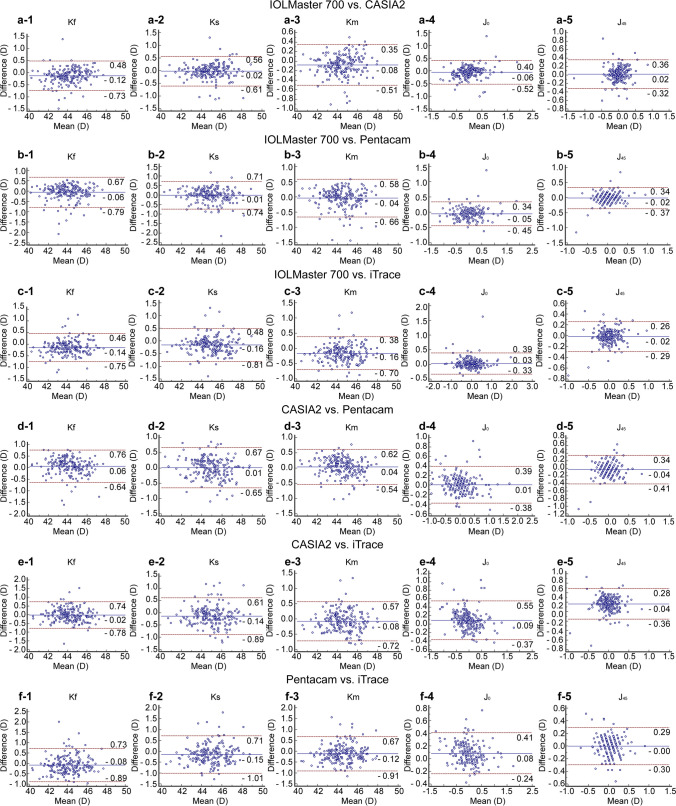
Bland–Altman plots comparing corneal parameters for IOLMaster 700, CASIA2, Pentacam and iTrace. Bland–Altman plots present the mean plotted against the differences in the values of Kf, Ks, Km, J_0_, J_45_ for comparisons between the IOLMaster 700 and CASIA2 (a-1 to a-5), IOLMaster 700 and Pentacam (b-1 to b-5), IOLMaster 700 and iTrace (c-1 to c-5), CASIA2 and Pentacam (d-1 to d-5), CASIA2 and iTrace (e-1 to e-5), and Pentacam and iTrace (f-1 to f-5). The blue solid line indicates the mean difference. The interval between the upper and lower dotted lines represents the 95% LoA

**Fig. 4:**
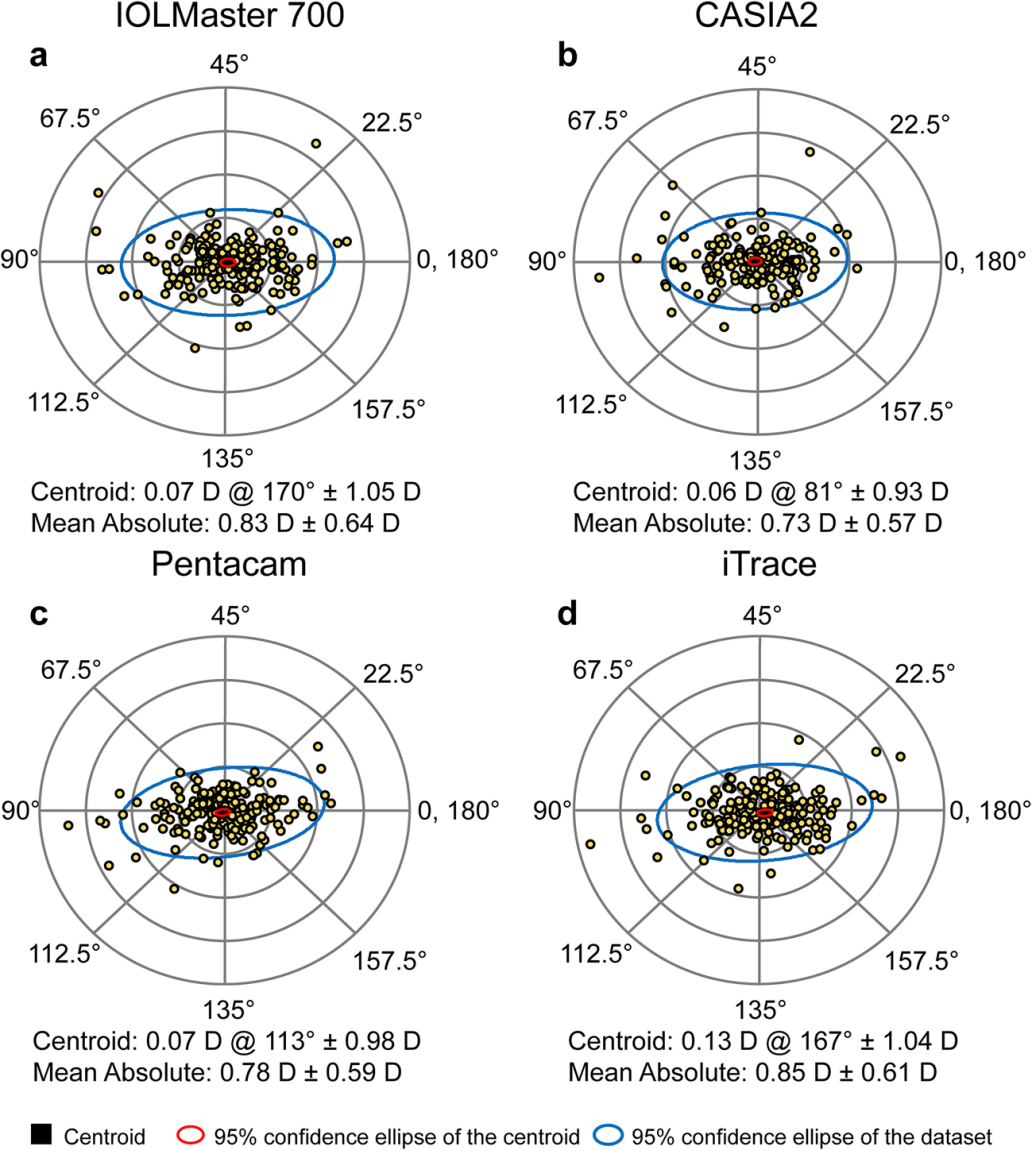
Double-angle plots of corneal astigmatism for IOLMaster 700, CASIA2, Pentacam and iTrace. Adjusted scale for each ring 1 d. The blue ellipse represents the 95% CI of the dataset. The red ellipse represents the 95% CI of the centroid

Table 2 shows the mean values and ranges for the measured parameters obtained using the four devices. Additionally, Figs. 1 and 2 show box plots and stacked histograms that more intuitively and visually demonstrate the distribution characteristics of the different measurement parameters. The Friedman test revealed that there were statistically significant differences between the four biometers in the Kf, Ks, Km, J_0_, and J_45_ (all *P* < 0.05). The post hoc analysis revealed that there were no significant differences between the IOLMaster 700 and Pentacam for any of the keratometry values (all *P* > 0.05). Additionally, there were no significant differences between the IOLMaster 700 and iTrace in evaluating J_0_ and J_45_ (all* P* > 0.05). Regarding the K values (Kf, Ks, Km), iTrace showed the largest mean value, followed by the CASIA2 and Pentacam, while the IOLMaster 700 gave the lowest value. The largest mean differences were found between the IOLMaster 700 and iTrace for Kf, Ks, and Km (0.14 D, 0.16 D, and 0.16 D, respectively). The Bland‒Altman plots revealed relatively large LoA widths for the K values, almost larger than one diopter. The smallest LoA width was observed between the IOLMaster 700 and CASIA2 for Km (0.860 D), while the largest was found between the Pentacam and iTrace for Ks (1.720 D) (Table 3 and Fig. 3). Regarding the power vectors, the J_0_ values were negative for the IOLMaster 700 and iTrace but positive for the CASIA2 and Pentacam. In terms of the absolute value of J_0_, iTrace had the largest value, while Pentacam had the smallest. The smallest LoA width was found between the Pentacam and iTrace for J_0_ (0.65 D). For J_45_, CASIA2 tended to indicate myopic oblique astigmatism, while the other devices had positive values. For the absolute value of J_45_, iTrace again had the largest value, while IOLMaster 700 and CAISA2 had the smallest values. Additionally, we can infer that the Pentacam tended to indicate positive values (Table 2). Figure 4 shows double angle plots for the IOLMaster 700, CASIA2, Pentacam and iTrace.

**Table 2:** Comparison of anterior corneal curvature measurements in cataract patients obtained with 4 devices

Value/Device	A	B	C	D	*P* value						
					4 device^※^	A vs B^*^	A vs C^*^	A vs D^*^	B vs C^*^	B vs D^*^	C vs D^*^
Kf, D											
Mean (SD)	44.04 (1.44)	44.16 (1.39)	44.10 (1.44)	44.18 (1.41)	< 0.001	< 0.001	1.0	< 0.001	< 0.001	0.701	< 0.001
Range	40.12, 47.66	40.31, 47.76	40.20, 47.90	40.44, 47.85							
Ks, D											
Mean (SD)	44.87 (1.45)	44.89 (1.43)	44.88 (1.49)	45.03 (1.45)	< 0.001	1.0	1.0	< 0.001	1.0	< 0.001	< 0.001
Range	40.36, 49.02	40.57, 48.78	40.20, 48.70	40.80, 48.84							
Km, D											
Mean (SD)	44.45 (1.41)	44.53 (1.38)	44.49 (1.44)	44.60 (1.40)	< 0.001	0.002	1.0	< 0.001	0.013	0.005	< 0.001
Range	40.24, 47.95	40.45, 47.95	40.20, 48.30	40.66, 48.03							
J_0_, D											
Mean (SD)	−0.03 (0.47)	0.03 (0.41)	0.02 (0.44)	−0.06 (0.47)	< 0.001	< 0.001	< 0.001	1.0	1.0	< 0.001	< 0.001
Range	−1.31, 2.06	−1.08, 1.73	−1.15, 1.69	−1.53, 1.82							
J_45_, D											
Mean (SD)	0.01 (0.25)	−0.01 (0.23)	0.02 (0.21)	0.03 (0.23)	0.018	0.847	1.0	1.0	0.027	0.060	1.0
Range	−1.36, 0.99	−1.28, 0.75	−0.74, 0.90	−0.81, 0.90							

*Kf* flat keratometry, *Ks* steep keratometry, *Km* mean keratometry, *J*_*0*_ (Jackson cross-cylinder at 0 and 90 degrees), *J*_*45*_ (Jackson cross-cylinder at 45 and 135 degrees), *D* diopter, *SD* standard error

A = IOLMaster 700, B = CASIA2, C = Pentacam, D = iTrace

^※^ *P* values calculated using the Friedman test

^*^ *P* values calculated using Bonferroni multiple-comparison test

**Table 3:** Agreement and correlation of anterior corneal curvature measurements obtained in patients with the four devices

Value	Mean difference (SD)	95% CI	95% LoA	LoA Width	ICC	95% CI	r	*P* value
Kf, D								
A vs. B	−0.12 (0.31)	−0.17, −0.08	−0.73, 0.48	1.21	0.972	0.955, 0.982	0.977	< 0.001
A vs. C	−0.06 (0.37)	−0.11, −0.01	−0.79, 0.67	1.46	0.966	0.955, 0.974	0.967	< 0.001
A vs. D	−0.14 (0.31)	−0.19, −0.10	−0.75, 0.46	1.21	0.972	0.948, 0.983	0.977	< 0.001
B vs. C	0.06 (0.36)	0.01, 0.11	−0.64, 0.76	1.40	0.967	0.957, 0.975	0.969	< 0.001
B vs. D	−0.02 (0.39)	−0.07, 0.03	−0.78, 0.74	1.52	0.961	0.949, 0.971	0.961	< 0.001
C vs. D	−0.08 (0.41)	−0.14, −0.02	−0.89, 0.73	1.62	0.957	0.942, 0.967	0.958	< 0.001
Ks, D								
A vs. B	−0.02 (0.30)	−0.06, 0.02	−0.61, 0.56	1.17	0.979	0.972, 0.984	0.979	< 0.001
A vs. C	−0.01 (0.37)	−0.06, 0.04	−0.74, 0.71	1.45	0.968	0.958, 0.976	0.969	< 0.001
A vs. D	−0.16 (0.33)	−0.21, −0.12	−0.81, 0.48	1.29	0.968	0.939, 0.981	0.974	< 0.001
B vs. C	0.01 (0.34)	−0.04, 0.06	−0.65, 0.67	1.32	0.973	0.965, 0.980	0.974	< 0.001
B vs. D	−0.14 (0.38)	−0.19, −0.09	−0.89, 0.61	1.50	0.960	0.939, 0.973	0.964	< 0.001
C vs. D	−0.15 (0.44)	−0.21, −0.09	−1.01, 0.71	1.72	0.951	0.927, 0.966	0.956	< 0.001
Km, D								
A vs. B	−0.08 (0.22)	−0.11, −0.05	−0.51, 0.35	0.86	0.986	0.978, 0.991	0.988	< 0.001
A vs. C	−0.04 (0.32)	−0.08, 0.004	−0.66, 0.58	1.24	0.975	0.967, 0.981	0.975	< 0.001
A vs. D	−0.16 (0.28)	−0.20, −0.12	−0.70, 0.38	1.08	0.974	0.941, 0.986	0.981	< 0.001
B vs. C	0.04 (0.29)	−0.001, 0.08	−0.54, 0.62	1.16	0.978	0.971, 0.983	0.979	< 0.001
B vs. D	−0.08 (0.33)	−0.12, −0.03	−0.72, 0.57	1.29	0.970	0.960, 0.978	0.972	< 0.001
C vs. D	−0.12 (0.40)	−0.17, −0.06	−0.91, 0.67	1.58	0.957	0.939, 0.969	0.960	< 0.001
J_0_, D								
A vs. B	−0.06 (0.24)	−0.09, −0.03	−0.52, 0.40	0.92	0.845	0.794, 0.884	0.861	< 0.001
A vs. C	−0.05 (0.20)	−0.08, −0.03	−0.45, 0.34	0.79	0.899	0.862, 0.926	0.906	< 0.001
A vs. D	0.03 (0.18)	0.003, 0.05	−0.33, 0.39	0.72	0.921	0.897, 0.940	0.923	< 0.001
B vs. C	0.01 (0.20)	−0.02, 0.03	−0.38, 0.39	0.77	0.890	0.857, 0.915	0.893	< 0.001
B vs. D	0.09 (0.23)	0.06, 0.12	−0.37, 0.55	0.92	0.841	0.763, 0.890	0.867	< 0.001
C vs. D	0.08 (0.17)	0.06, 0.11	−0.24, 0.41	0.65	0.920	0.845, 0.953	0.937	< 0.001
J_45_, D								
A vs. B	0.02 (0.17)	−0.004, 0.04	−0.32, 0.36	0.68	0.727	0.654, 0.786	0.731	< 0.001
A vs. C	−0.02 (0.18)	−0.04, 0.01	−0.37, 0.34	0.71	0.696	0.617, 0.761	0.704	< 0.001
A vs. D	−0.02 (0.14)	−0.04, 0.002	−0.29, 0.26	0.55	0.823	0.772, 0.863	0.827	< 0.001
B vs. C	−0.04 (0.19)	−0.06, −0.01	−0.41, 0.34	0.75	0.604	0.507, 0.686	0.612	< 0.001
B vs. D	−0.04 (0.16)	−0.06, −0.01	−0.36, 0.28	0.64	0.727	0.648, 0.789	0.736	< 0.001
C vs. D	−0.001 (0.15)	−0.02, 0.02	−0.30, 0.29	0.59	0.761	0.696, 0.814	0.762	< 0.001

*Kf* flat keratometry, *Ks* steep keratometry, *Km* mean keratometry, *D* diopter, *SD* standard error, *CI* confidence interval, *LoA* limits of agreement, *ICC* intraclass correlation coefficient, *r* pearson coefficient

A = IOLMaster 700, B = CASIA2, C = Pentacam, D = iTrace

The Pearson coefficients indicated strong correlations between all pairs of devices (all r > 0.6) (Table 3) [13]. The ICCs (with 95% CIs) for Kf (0.966; 0.957–0.973), Ks (0.966; 0.957–0.974), and Km (0.973; 0.966–0.979) indicated excellent agreement among the four devices. The ICCs (with 95% CIs) for J_0_ (0.887; 0.858–0.911) showed good to excellent agreement. However, the ICCs (with 95% CIs) for J_45_ (0.725; 0.673–0.773) showed moderate to good agreement among the SS-OCT-based devices, the Scheimpflug system and the ray-tracing aberrometer (Table 4).

**Table 4:** ICC calculation among the 4 devices

Value	ICC	95% CI		F test with true value 0			
		Lower bound	Upper bound	Value	Df1	Df2	*P*
Kf, D	0.966	0.957	0.973	121.273	199	597	< 0.001
Ks, D	0.966	0.957	0.974	125.687	199	597	< 0.001
Km, D	0.973	0.966	0.979	159.987	199	597	< 0.001
J_0_, D	0.887	0.858	0.911	35.216	199	597	< 0.001
J_45_, D	0.725	0.673	0.773	11.708	199	597	< 0.001

*Kf* flat keratometry, *Ks* steep keratometry, *Km* mean keratometry, *D* diopter, *ICC* intraclass correlation coefficient, *CI* confidence interval

Further analysis was conducted on the differences in posterior corneal (PK) and total corneal (TK) data between the IOLMaster700, CASIA2, and Pentacam, as well as the comparison between the total corneal (TK) of IOLMaster700 and the true net power (TNP) of CASIA2 and Pentacam. As iTrace cannot measure posterior surface data, it was not included in this comparison (Table 5).

**Table 5:** Comparison of posterior keratometry and total keratometry measurements by 3 devices

Value/Device	A	B	C	*P* value							
				3 device^※^	A vs B^*^	A vs C^*^		B vs C^*^	ICC		*P*
PKf, D											
Mean (SD)	−5.84 (0.23)	−6.15 (0.22)	−6.34 (0.24)	< 0.001	< 0.001	< 0.001		< 0.001	0.419		< 0.001
Range	−6.38, −5.14	−6.6, −5.4	−6.9, −5.5								
PKs, D											
Mean (SD)	−6.07 (0.25)	−6.42 (0.26)	−6.63 (0.28)	< 0.001	< 0.001	< 0.001		< 0.001	0.426		< 0.001
Range	−6.88, −5.17	−7.2, −5.5	−7.6, −5.6								
PKm, D											
Mean (SD)	−5.95 (0.23)	−6.27 (0.23)	−6.46 (0.24)	< 0.001	< 0.001	< 0.001		< 0.001	0.429		< 0.001
Range	−6.57, −5.15	−6.9, −5.4	−7.2, −5.5								
PK-J_0_, D											
Mean (SD)	−0.10 (0.07)	−0.13 (0.07)	−0.11 (0.10)	< 0.001	< 0.001	< 0.05		< 0.001	0.637		< 0.001
Range	−0.33, 0.09	−0.30, 0.05	−0.40, 0.30								
PK-J_45_, D											
Mean (SD)	−0.02 (0.05)	0.01 (0.04)	−0.01 (0.06)	< 0.001	< 0.001		0.916	< 0.001		0.553	< 0.001
Range	−0.18, 0.14	−0.14, 0.12	−0.20, 0.22								
TKf, D											
Mean (SD)	44.03 (1.44)	43.07 (1.35)	44.01 (1.51)	< 0.001	< 0.001	0.329		< 0.001	0.830		< 0.001
Range	40.34, 47.73	39.30, 46.5	39.7, 48.5								
TKs, D											
Mean (SD)	44.93 (1.44)	44.84 (1.40)	44.88 (1.57)	< 0.001	< 0.001	< 0.05		< 0.001	0.814		< 0.001
Range	40.62, 48.84	39.70, 47.60	40.30, 49.00								
TKm, D											
Mean (SD)	44.47 (1.40)	43.44 (1.34)	44.45 (1.51)	< 0.001	< 0.001	0.121		< 0.001	0.821		< 0.001
Range	40.48, 47.88	39.50, 46.75	40.00, 48.80								
TK-J_0_, D											
Mean (SD)	−0.13 (0.48)	−0.09 (0.41)	−0.01 (0.48)	< 0.001	0.137	< 0.001		< 0.001	0.826		< 0.001
Range	−1.46, 2.02	−1.25, 1.63	−1.26, 2.24								
TK-J_45_, D											
Mean (SD)	−0.01 (0.25)	−0.005 (0.23)	0.01 (0.25)	0.787	> 0.05	> 0.05		> 0.05	0.686		< 0.001
Range	−1.43, 0.97	−1.32, 0.79	−0.77, 0.99								

*PK* posterior keratometry, *TK* total keratometry, *Kf* flat keratometry, *Ks* steep keratometry, *Km* mean keratometry, *J0* Jackson cross-cylinder at 0 and 90 degrees, *J45* Jackson cross-cylinder at 45 and 135 degrees, *D* diopter, *SD* standard error, *ICC* intraclass coefficient of correlation

A = IOLMaster 700, B = CASIA2, C = Pentacam

^※^ *P* values calculated using the Friedman test

^*^ *P* values calculated using Bonferroni multiple-comparison test

Significant differences were found in PK values, PK astigmatism, TK values, and TK astigmatism among the three devices (all *P*<0.001). It's noteworthy that the IOLMaster 700 reported the highest TK values and the lowest PK values among the three devices, and these measurements were not interchangeable due to their poor consistency.

Significant differences were also found among the total corneal (TK) of IOLMaster700 and the true net power (TNP) of CASIA2 and Pentacam (*P* < 0.001). The IOLMaster 700's TK value was the highest, followed by the TNP value of Pentacam, with the TNP value of CASIA2 being the smallest. The correlation between the TK/TNP values of the three devices was not high (ICC = 0.142, *P* < 0.001), suggesting that they cannot be used interchangeably.

## Discussion

Measuring corneal curvature and astigmatism is essential in cataract patients, as it can help to determine the appropriate intraocular lens power required for surgery. In a study of 23,239 eyes, a corneal astigmatism of 1.0 diopter or more was observed in approximately one-third of patients [14]. Another study of 13,012 eyes of 6,506 patients revealed that 43.5% of eyes had an astigmatism > 1.00 D [15]. Accurate measurements of these parameters are crucial for achieving optimal visual outcomes, minimizing refractive errors, and improving patient satisfaction. Additionally, it can assist in identifying preexisting conditions such as keratoconus or other corneal abnormalities that may affect surgical outcomes. The selection of the appropriate device for measuring corneal curvature and astigmatism is critical, as different devices may provide varying degrees of accuracy and precision.

In this study, we compared the measurements of corneal curvature and corneal astigmatism in 200 cataract patients using four different devices: an IOLMaster 700, CASIA2, Pentacam, and iTrace. Previous studies [16–20] have mainly focused on comparing two or three of these four devices. To our knowledge, this is the first study to date to analyze data using all four devices. While our findings indicated strong correlations and excellent agreement among the IOLMaster 700, CASIA2, Pentacam, and iTrace in measuring anterior corneal curvature and astigmatism, further analysis revealed significant differences among their measurements. These differences suggest that while the devices are generally accurate in their measurements, their precision and reliability can vary significantly. Therefore, despite their overall accuracy, caution should be exercised when using these devices interchangeably due to potential variations in their precision and reliability.

In the current study, the iTrace provided a steeper corneal curvature than the SS-OCT devices and Scheimpflug camera system. Similarly, for the absolute values of J_0_ and J_45_, the iTrace also yielded the largest values. In contrast, Park et al. [21] demonstrated that IOLMaster provided steeper keratometric values than the iTrace device. Whang et al. [22] also found that the K-readings of the IOLMaster were higher than those of Pentacam. Notably, the authors of that study used IOLMaster 500. No significant differences were observed between any of the keratometry values obtained by the IOLMaster 700 and those obtained by the Scheimpflug imaging system or between the IOLMaster 700 and iTrace for J_0_ and J_45_. However, the Bland‒Altman analysis revealed relatively large LoA widths. For all pairwise comparisons of K-values among the four devices, the smallest LoA width was greater than 0.8 D, and the largest was nearly 2 D. A 0.5 D difference in the corneal plane causes a difference of approximately 0.73 D in the IOL plane [23]. In other words, a difference of approximately 0.8 D in the corneal plane causes a difference of more than 1 D in the IOL plane. For J_0_ and J_45_, the smallest LoA width was larger than 0.5 D among the four devices. An astigmatism correction of more than 0.5 D improves the visual outcomes of cataract surgery [24].

Differences in the optical zones evaluated by the devices and the technologies used to measure the parameters could partly explain the observed results. Specifically, the IOLMaster 700 obtained keratometry for a diameter of 2.5 mm, while the Pentacam and iTrace calculated corneal power for a 3.0 mm optical zone, and the CASIA2 used a measuring diameter of 3.2 mm. Given that different parts of the cornea may have slightly different curvatures, these variations in measurement areas can lead to disparities in corneal power measurements. Moreover, the different measurement technologies used by these devices, namely SS-OCT for the IOLMaster 700 and CASIA2, a Scheimpflug camera system for the Pentacam, and a ray-tracing aberrometer for the iTrace, can also contribute to the variation in measurements due to their different measurement principles and techniques [25–28].

Despite the strong correlation and good agreement among the IOLMaster700, CASIA2, Pentacam, and iTrace for Kf, Ks, Km and J0, J45, significant differences and considerable variations were observed among the measurements from these devices. These differences can be attributed to the different technologies used by these devices and the different optical zones they measure. Therefore, while these devices provide similar measurements of corneal power, they should not be used interchangeably due to the larger LoA widths and the presence of significant differences. Clinicians should be cautious when interpreting these measurements, particularly when making decisions about treatment options such as the selection of intraocular lens power for cataract surgery.

Our study further explored the differences and comparisons in the data of the posterior corneal surface (PK) and total corneal power (TK) measured by IOLMaster700, CASIA2, and Pentacam. We found that among the three devices, IOLMaster 700 measured the largest TK value, while CASIA2 measured the smallest. Pentacam measured the largest PK value, while IOLMaster 700 measured the smallest. The differences among TK and PK values measured by the three devices were significant, and the consistency was poor, suggesting that they should not be used interchangeably. Furthermore, we found that the average TK value measured by IOLMaster 700 was the largest, at 44.47D, followed by the average TNP value measured by Pentacam, at 43.12D, and the smallest average TNP value measured by CASIA2, at 38.36D. The differences in TK/TNP values among IOLMaster 700, CASIA2, and Pentacam were significant, and the correlation among them was low (ICC = 0.142, *P* < 0.001), suggesting that they should not be used interchangeably. Importantly, our study has several limitations. Firstly, our study only included patients with cataracts, which may limit the generalizability of our findings to other populations. Secondly, our study focused on comparing preoperative measurements from the IOLMaster 700, CASIA2, Pentacam, and iTrace devices. However, we did not assess or discuss the accuracy of these measurements in terms of refractive outcomes due to the absence of documented postoperative results. While the repeatability, reliability, and accuracy of these devices have been previously reported, the ultimate aim of these measurements is to improve refractive outcomes for patients. Therefore, the lack of comparison of the predictive outcomes of these devices with actual postoperative results is a limitation. Future studies are needed to address this issue and provide further insights into the clinical utility of these devices.

Selecting the most appropriate device is essential to ensure optimal surgical outcomes for individual patients. For routine screening without posterior corneal data requirements, the IOLMaster700 or CASIA2 OCT devices provide good accuracy balanced with affordability. However, for premium IOL calculations or post-refractive surgery cases, Scheimpflug and ray-tracing technologies such as the Pentacam and iTrace are recommended to account for posterior corneal measurements. Availability of historical records, specific corneal conditions, and cost constraints should also help guide optimal device choice for the clinical context.

## Conclusions

Measuring corneal curvature and astigmatism accurately is crucial for achieving optimal visual outcomes in cataract surgery. Our study found that all four devices (IOLMaster 700, CASIA2, Pentacam, and iTrace) are capable of accurately measuring these parameters, but there are some differences in the precision and reliability of the measurements. Therefore, caution should be taken when interpreting measurements obtained from different devices, as they may provide significantly different results. It is important to choose the appropriate device for each patient to ensure the best possible surgical outcome. Further studies are needed to assess the impact of these differences on postoperative outcomes.

## Data Availability

The datasets used during the current study are available from the corresponding author on reasonable request.
